# Optimization of Medium Using Response Surface Methodology for Lipid Production by *Scenedesmus* sp.

**DOI:** 10.3390/md12031245

**Published:** 2014-03-06

**Authors:** Fangfang Yang, Lijuan Long, Xiumei Sun, Hualian Wu, Tao Li, Wenzhou Xiang

**Affiliations:** 1 Key Laboratory of Tropical Marine Bio-resources and Ecology, South China Sea Institute of Oceanology, Chinese Academy of Sciences, Guangzhou 510301, China; E-Mails: ycuyang@163.com (F.Y.); longlj@scsio.ac.cn (L.L.); sunxm@scsio.ac.cn (X.S.); hlwu@scsio.ac.cn (H.W.); taoli@scsio.ac.cn (T.L.); 2 Graduate School of Chinese Academy of Sciences, Beijing 100049, China

**Keywords:** optimization, lipid production, *Scenedesmus* sp., response surface methodology, biodiesel

## Abstract

Lipid production is an important indicator for assessing microalgal species for biodiesel production. In this work, the effects of medium composition on lipid production by *Scenedesmus* sp. were investigated using the response surface methodology. The results of a Plackett–Burman design experiment revealed that NaHCO_3_, NaH_2_PO_4_·2H_2_O and NaNO_3_ were three factors significantly influencing lipid production, which were further optimized by a Box–Behnken design. The optimal medium was found to contain 3.07 g L^−1^ NaHCO_3_, 15.49 mg L^−1^ NaH_2_PO_4_·2H_2_O and 803.21 mg L^−1^ NaNO_3_. Using the optimal conditions previously determined, the lipid production (304.02 mg·L^−1^) increased 54.64% more than that using the initial medium, which agreed well with the predicted value 309.50 mg L^−1^. Additionally, lipid analysis found that palmitic acid (C16:0) and oleic acid (C18:1) dominantly constituted the algal fatty acids (about 60% of the total fatty acids) and a much higher content of neutral lipid accounted for 82.32% of total lipids, which strongly proved that *Scenedesmus* sp. is a very promising feedstock for biodiesel production.

## 1. Introduction

In recent years, since the energy crisis and climate change have been major challenges we are facing, it is essential to develop novel energy forms, which are sustainable and friendly to the environment [1,2]. As an ideal and effective alternative fuel, biofuel has drawn more and more attention of researchers. To date, three generations of biofuel feedstocks have been developed [3]. Compared to the first and second generation (food crops, non-food crops), microalgae, a third generation biofuel feedstock, have been indicated as a superior replacement, because of their capability to grow rapidly and produce abundant triacylglycerols (TAG). Moreover, microalgae can survive over a wide range of environmental conditions, even non-arable land and saline water. Therefore, producing oils by microalgae does not result in a discord between food and fuel [4,5,6].

Based upon these advantages of microalgae, the annual lipid yield can achieve 200 barrels per hectare of land in theory [7]. So far, producing biodiesel by microalgae has obtained significant advances in small-scale laboratory experiments and field testing stages [7,8]. However, due to the high cost and low lipid yield, microalgae-based biodiesel production still lacks economic viability at a large-scale. Therefore, optimization of lipid production is important for biodiesel production from microalgae. Extensive research revealed that environmental conditions can modify the lipid metabolism of microalgae efficiently [9,10,11]. In particular, nutritional factors (e.g., nitrogen, phosphorus, carbon and iron) are recognized as one of the most crucial factors influencing the lipid accumulation and the yield of biomass [12,13,14,15,16]. Generally, nutrient starvation, such as nitrogen and phosphorus deficiency, can stimulate lipid accumulation for several microalgae species [17,18]. For instance, the lipid content of *Nannochloris* sp. UTEX LB1999 had an 83.08% increase with the nitrogen concentration decreasing to 0.9 mM [19]. However, the deficiencies in a few nutrients have been also observed to severely limit the growth of microalgae. As a result, the overall lipid production, which is the product of the growth rate multiplied by the lipid content, may be lower [13,20]. Moreover, these studies have been carried out to investigate single-factor optimization. It is obvious that the classical method of optimization may bring about unsatisfactory or incorrect results, due to the ignoring of the interaction between factors.

The response surface methodology (RSM) is an effective and convenient method for screening key factors rapidly from multiple factors and optimizing culture conditions, which can avoid the defects brought by single-factor optimization [21,22]. The method has already been successfully utilized in many fields, such as the chemical industry, engineering, biology, *etc*. [23,24,25]. However, only a few reports related the application of RSM in the optimizing of autotrophic microalgal medium for lipid production, where RSM had been shown to enhance lipid production by a two-step strategy with initial optimization of microalgal growth and final optimization of lipid accumulation [26,27]. To our knowledge, the research using directly RSM for improving the value of lipid production by one-stage culture in the autotrophic microalgae has been scarcely reported till now. Additionally, differences among species exist and, sometimes, even among strains of the same species. Therefore, for each individual microalga, systematic studies are needed to optimize the medium in order to obtain its maximum lipid production.

In this study, a green microalga identified as *Scenedesmus* sp. was isolated. Due to its strong tolerance to high alkalinity and salinity, this species could be resistant to contamination by other organisms. In our previous experiment, the feasibility of *Scenedesmus* sp. culture has been demonstrated in an outdoor raceway system of up to 66 m^3^. However, the lipid production needed to be improved further. To maximize its lipid production, a Plackett–Burman design was used to evaluate the significance of nine nutrient factors of the medium towards lipid production, and then, a Box–Behnken design was also utilized to identify the best culture strategy. Furthermore, the extracted lipids were investigated to further assess the potential of *Scenedesmus* sp. in producing biodiesel.

## 2. Results and Discussion

### 2.1. Evaluating the Significant Nutrient Factors Using Plackett–Burman Design

The Plackett–Burman design with two coded levels for all twelve runs was employed to analyze comprehensively the influence of nine nutrient components on the response value-lipid production. The lipid production was the product of lipid content and biomass, the importance of which is above the lipid content and growth rate individually. Therefore, lipid production was a reliable indicator for evaluating algal species for biodiesel production [20]. The experimental data, illustrated in Table 1 and Supplementary Table S1, were calculated by the Design-Expert software, and the results of variance analysis and the estimation of parameters are listed in Table 2. The *p*-value was used to evaluate the significance of the variable. When the *p-*value of the variable was less than 5%, it represented that the variable had significant effects on the response value. To further assess the effect of the variable, coefficient estimate was applied. Lipid production could grow with increasing concentrations of the variable if the coefficient estimate were positive; conversely, the value was negative, indicating that lipid production was negatively correlated with the variable levels [28]. As shown in Table 2, A_5 _solution, soil extract and NaH_2_PO_4_·2H_2_O had a negative effect, whereas the other factors displayed a positive effect on lipid production. NaH_2_PO_4_·2H_2_O was the most important variable impacting lipid production and growth, with *p-*value less than 0.0001. With decreasing phosphate concentrations from 100 mg L^−1^ to 25 mg L^−1^, the cellular lipid content in microalgae *Scenedesmus* sp. increased evidently, where the *p*-value was less than 0.001. Furthermore, low phosphate had a positive effect on biomass associated with inducing a higher lipid accumulation in cells. Therefore, lipid production was observed to be more with the low phosphate medium than with the high phosphate medium (Table 2).

So far, various studies have been carried out to demonstrate that the nitrogen source was the important nutrition in the medium affecting the growth and lipid accumulation [13]. There was evidence to suggest that nitrogen deficiency could stimulate lipid accumulation [29,30]. However, under the applied experimental conditions, this phenomenon was not observed; instead, NaNO_3_ had a positive effect on lipid production (Table 2). The reason might be that the NaNO_3_ concentration did not reach the limiting level for *Scenedesmus* sp. In fact, these experiments were limited to nine days of culture, and the nitrogen level was set in the range of 250 and 1,000 mg·L^−1^, which was higher than that previously reported [29]. In a short period of time, the NaNO_3_ may not be depleted and reach the limiting level. Additionally, although the lipid production was increased with the increasing nitrogen level, the contribution of NaNO_3_ was low, with 4.47%.

**Table 1:** Results and experimental layout of *Scenedesmus* sp. in a Plackett–Burman design.

Run	X_1_	X_2_	X_3_	X_4_	X_5_	X_6_	X_7_	X_8_	X_9_	Lipid production (mg·L^−1^)
1	4	200	25	1000	40	100	0.5	0.5	0.5	230.38
2	1	200	100	250	40	100	2	0.5	0.5	174.27
3	4	50	100	1000	10	100	2	2	0.5	188.56
4	1	200	25	1000	40	20	2	2	2	208.85
5	1	50	100	250	40	100	0.5	2	2	152.85
6	1	50	25	1000	10	100	2	0.5	2	199.58
7	4	50	25	250	40	20	2	2	0.5	215.37
8	4	200	25	250	10	100	0.5	2	2	214.41
9	4	200	100	250	10	20	2	0.5	2	173.23
10	1	200	100	1000	10	20	0.5	2	0.5	166.16
11	4	50	100	1000	40	20	0.5	0.5	2	188.82
12	1	50	25	250	10	20	0.5	0.5	0.5	196.58

X_1_, NaHCO_3_ (g·L^−1^); X_2_, KCl (mg·L^−1^); X_3_, NaH_2_PO_4_·2H_2_O (mg·L^−1^); X_4_, NaNO_3_ (mg·L^−1^); X_5_, CaCl_2_ (mg·L^−1^); X_6_, MgSO_4_·7H_2_O (mg·L^−1^); X_7_, EDTA-Fe^3+^ (mL·L^−1^); X_8_, A_5_ solution (mL·L^−1^); X_9_, soil extract (mL·L^−1^).

**Table 2:** Statistical analysis of the Plackett–Burman experiment design.

Factor	Level	Effect	Sum of Squares	Contribution %	Coefficient Estimate	*t*-value	*p*-value	Effect
	−1 +1							
NaHCO_3_	1	4	18.7476	1,054.3100	18.2703	9.37	0.0034	0.0015 ^a^
KCl	50	200	4.2567	54.3576	0.9420	1.9608	0.1537	—
NaH_2_PO_4_·2H_2_O	25	100	−36.8800	4080.4000	70.7096	−18.6075	0.0006	<0.0001 ^a^
NaNO_3_	250	1000	9.2733	257.9840	4.4706	4.4692	0.0582	0.0477 ^a^
CaCl_2_	10	40	5.3367	85.4400	1.4806	2.8358	0.1306	—
MgSO_4_·7H_2_O	20	100	1.8400	10.1568	0.1760	1.0875	0.3203	—
EDTA-Fe^3+^	0.5	2	1.7767	9.4696	0.1641	1.0558	0.2877	—
A_5_	0.5	2	−2.7767	23.1296	0.4008	−1.5558	0.1907	—
Soil extract	0.5	2	−5.5967	93.9680	1.6284	−2.6308	0.1046	—

^a^ Significance level at a *p*-value less than 5%; —, significance level at a *p*-value of more than 5%, *R*^2^ = 0.9345; *R*_adj_^2^ = 0.9099; *R*_pred_^2^ = 0.8526; coefficient of variation (CV) = 3.57.

In this study, NaHCO_3_ was also identified as a significant factor for lipid production. It was obvious that increasing the concentration of carbon could dramatically promote the growth rate of *Scenedesmus* sp. (*p*-value lower than 0.0001). The lipid production was improved considerably with increasing carbon concentration, which accounted for 18.27% of the total contribution (Table 2). This was in agreement with previous reports [16,31,32]. For instance, the lipid production was significantly increased when supplemented with 2 g·L^−1^ bicarbonate, compared with zero and 1 g·L^−1^ bicarbonate in microalga *Tetraselmis suecica* and *Nannochlorpsis salina* [16]. Besides, growing well in a high level of NaHCO_3_ (3 g·L^−1^) implied that *Scenedesmus* sp. had a high tolerance for alkalinity.

In conclusion, NaH_2_PO_4_·2H_2_O, NaHCO_3_ and NaNO_3_ were the important variables impacting lipid production, whereas other factors were insignificant, suggesting that they were not limiting in the process of lipid production. Therefore, NaH_2_PO_4_·2H_2_O, NaHCO_3_ and NaNO_3_ were chosen to make further optimization by the Box–Behnken design.

In order to check the fit of the model, *R*^2^ and *F*-value were calculated. Here, *R*^2^ was 0.9345, indicating that 93.45% of the data in Plackett–Burman design could be explained by the model; that is, the proposed model was reasonable. Moreover, the model *F*-value of 38.05 demonstrated that the model was significant, as revealed by a *p*-value lower than 0.0001, which further supported that the model was fit to these data. From the analysis of *R*_adj_^2^ and *R*_pred_^2^, the *R*_pred_^2^ of 0.8526 was in good agreement with the *R*_adj_^2^ of 0.9099 (Table 2). In conclusion, the model was used to explain the data well.

### 2.2. Identifying the Best Culture Conditions for Lipid Production Using Box–Behnken Design

Based on the results of the previous experiments, the Box–Behnken design was used to further confirm the optimum concentrations of NaH_2_PO_4_·2H_2_O, NaHCO_3_ and NaNO_3_, to maximize lipid production. In this experiment, five replicates of the center points and twelve star points were required, resulting in a total number of seventeen experiments. Table 3 presented the experimental project and the experimental and predicted values of response. Among the seventeen experiments, experiment seventeen (NaHCO_3_, NaH_2_PO_4_·2H_2_O and NaNO_3_ concentrations of 3 g L^−1^, 15 mg L^−1^, 750 mg L^−1^) offered the highest lipid production (315.74 mg L^−1^), while experiment five (NaHCO_3_, NaH_2_PO_4_·2H_2_O and NaNO_3_ concentrations of 2 g L^−1^, 15 mg L^−1^, 500 mg L^−1^) provided the lowest total lipid production (171.96 mg L^−1^).

**Table 3:** Experimental design and lipid production in the Box-Behnken design.

Run	NaHCO_3_ (g L^−1^)	NaH_2_PO_4_·2H_2_O (mg L^−1^)	NaNO_3_ (mg L^−1^)	Lipid production (mg L^−1^)	
				Experimental	Predicted
1	2	10	750	207.53	207.77
2	4	10	750	211.29	209.08
3	2	20	750	186.23	188.44
4	4	20	750	229.44	229.20
5	2	15	500	171.96	166.17
6	4	15	500	201.35	198.01
7	2	15	1000	203.78	207.12
8	4	15	1000	211.58	217.37
9	3	10	500	240.55	246.1
10	3	20	500	200.97	204.55
11	3	10	1000	237.89	234.31
12	3	20	1000	282.19	276.64
13	3	15	750	304.86	307.51
14	3	15	750	305.22	307.51
15	3	15	750	310.39	307.51
16	3	15	750	301.35	307.51
17	3	15	750	315.74	307.51

By applying multiple regression analysis on the data above, the equation for lipid production was established as follows:

Lipid production = −784.5075 + 423.4910NaHCO_3_ + 14.9340NaH_2_PO_4_·2H_2_O + 0.8162NaNO_3_ + 1.9725NaHCO_3_*NaH_2_PO_4_·2H_2_O − 0.0216NaHCO_3_*NaNO_3_ + 0.0168NaH_2_PO_4_·2H_2_O*NaNO_3_ − 71.0610NaHCO_3_^2^ − 1.1131 NaH_2_PO_4_·2H_2_O^2^ − 0.0006NaNO_3_^2^.

In order to investigate the adequacy of the model, multiple regression analyses on the data were applied. The results are listed in Table 4, which were mainly the individual effects of all variables and their interactions on lipid production. The multiple correlation coefficient *R*^2^ of 0.992 suggested that the quadratic polynomial model was suitable for revealing the mutual relationship of factors and predicting the response values in the study (Table 4).

As shown in Table 4, NaHCO_3_ and NaNO_3_ exerted significant individual and quadratic effects, respectively (*p*-value less than 0.05). NaH_2_PO_4_·2H_2_O, varying from 10 mg L^−1^ to 20 mg L^−1^, was not significant (*p*-value more than 0.05), yet with significant quadratic effects for the response value (*p*-value less than 0.05).

**Table 4:** Statistical analysis of the Box−Behnken experiment design.

Factor	Sum of squares	Degree of Freedom	Mean square	Coefficient Estimate	*F*-value	*p*-value
Model	38,929.9101	9	4,325.5456	307.5120	96.8736	<0.0001
NaHCO_3_	885.3632	1	885.3632	10.5200	19.8283	0.0030
NaH_2_PO_4_·2H_2_O	0.3081	1	0.3081	0.1963	0.0069	0.9361
NaNO_3_	1,818.3465	1	1,818.3465	15.0763	40.7231	0.0004
NaHCO_3_*NaH_2_PO_4_·2H_2_O	389.0756	1	389.0756	9.8625	8.7136	0.0213
NaHCO_3_*NaNO_3_	116.5320	1	116.5320	−5.3975	2.6098	0.1502
NaH_2_PO_4_·2H_2_O*NaNO_3_	1,758.9636	1	1,758.9396	20.9700	39.3932	0.0004
NaHCO_3_^2^	21,261.7504	1	21,261.7504	−71.0610	476.1715	<0.0001
NaH_2_PO_4_·2H_2_O^ 2^	3,260.7386	1	3,260.7386	−27.8285	73.0265	<0.0001
NaNO_3_^2^	6,497.6563	1	6,497.6563	−39.2835	145.5195	<0.0001
Residual	312.5602	7	44.6515			
Lack of fit	186.3207	3	62.1069		1.9679	0.2609
Pure error	126.2395	4	31.5599			
Corr. total	39,242.4703	16				
Model	38,929.9101	9	4,325.5456	307.5120	96.8736	<0.0001

*R*^2^ = 0.9920; *R*_adj_^2^ = 0.9818; *R*_pred_^2^ = 0.9190; coefficient of variation (CV) = 2.76.

**Figure 1:**
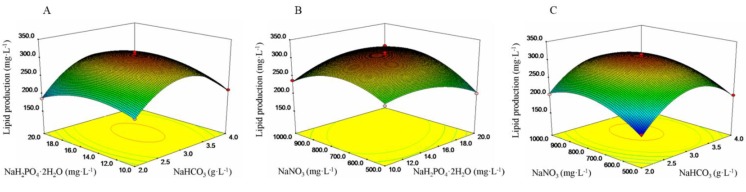
Three-dimensional response surface plots for lipid production showing the interactions effects of (**A**) NaHCO_3_ and NaH_2_PO_4_·2H_2_O; (**B**) NaH_2_PO_4_·2H_2_O and NaNO_3_; and (**C**) NaHCO_3_ and NaNO_3_.

The interactions between three parameters (NaHCO_3_, NaH_2_PO_4_·2H_2_O and NaNO_3_) and lipid production were revealed by response surface plots and contour plots, as shown in Figure 1. Figure 1A represents the effects of NaHCO_3_ and NaH_2_PO_4_·2H_2_O levels individually and their mutual interaction on the lipid production. Varying NaHCO_3_ and NaH_2_PO_4_·2H_2_O concentration mutual interactions had a significant effect on the total lipid production value. The increase in NaHCO_3_ and NaH_2_PO_4_·2H_2_O concentrations enhanced the production of lipid initially, but then, with increasing their concentrations further, which exceed 3.07 and 15.49 mg L^−1^, respectively, the lipid production could decrease. The highest response value was observed at 3.07 g L^−1^ NaHCO_3_ and 15.48 mg L^−1^ NaH_2_PO_4_·2H_2_O (Figure 1A). A similar phenomenon was observed in Figure 1B with NaH_2_PO_4_·2H_2_O and NaNO_3_ while maintaining other variables constant. However, the lipid production was almost constant when NaHCO_3_ and NaNO_3_ concentrations were increased at a fixed NaH_2_PO_4_·2H_2_O concentration (Figure 1C). This implied that the interaction between NaHCO_3_ and NaNO_3_ did not have a significant effect on lipid production under nitrogen sufficiency.

According to the attained results and the equation, the model predicted the maximum lipid production of 309.50 mg L^−1^ in the concentration of 3.07 g L^−1^ NaHCO_3_, 15.49 mg L^−1^ NaH_2_PO_4_·2H_2_O and 803.21 mg L^−1^ NaNO_3_. The final optimum condition was as follows: NaHCO_3_, 3.07 g L^−1^; NaH_2_PO_4_·2H_2_O, 0.01549 g L^−1^; NaNO_3_, 0.80321 g L^−1^; CaCl_2_, 0.02 g L^−1^; MgSO_4_·7H_2_O, 0.05 g L^−1^; KCl, 0.1 g L^−1^; A_5_, 1 mL L^−1^; EDTA-Fe^3+^, 1 mL L^−1^; soil extract, 1 mL L^−1^. Under the optimum condition, the biomass and lipid content were 0.93 g·L^−1^ and 32.69% dry weight (dw), which were increased 13.41% and 36.32% more than those of the original condition, respectively. The observed lipid production was 304.02 mg·L^−1^, agreeing well with the predicted value, indicating that the model was valid. Compared with that under the original culture condition, lipid production increased 54.64%.

### 2.3. Lipid Analysis and Fatty Acid Composition

The lipid profiles and fatty acid composition of *Scenedesmus* sp. were studied to evaluate the optimum medium in terms of lipid quality. As shown in Table 5, whatever the culture conditions applied, the fatty acid composition of *Scenedesmus* sp. was similar. The dominant fatty acids included palmitic acid (C16:0), oleic acid (C18:1), linoleic acid (C18:2) and linolenic acid (C18:3), accounting for about 90% of the total fatty acids. Demirbas and Demirbas [33] reported that C16:0 and C18:1 were the most important fatty acids, which were considered as the indicators for the quality of biodiesel. In the study, we observed that the C16:0 and C18:1 presented in major quantities (about 60% of the total fatty acids), implying that *Scenedesmus* sp. was suitable for biodiesel production. Similar results were reported by Chen *et al*. [34].

**Table 5:** Fatty acid composition of *Scenedesmus* sp. (a) in the original medium; (b) in the optimized medium. Values were given as the means of total FAME percentage ± standard deviation.

Fatty acid (%)	C16:0	C16:1	C16:2	C18:0	C18:1	C18:2	C18:3
a	29.43 ± 1.75	1.99 ± 0.69	2.42 ± 0.24	7.33 ± 0.13	30.04 ± 1.02	13.60 ± 0.45	12.76 ± 0.26
b	30.77 ± 0.76	1.96 ± 0.54	1.37 ± 0.02	3.41 ± 0.09	36.27 ± 0.78	11.55 ± 0.12	12.52 ± 0.39

In the study, although no significant differences in fatty acid composition were observed, it was obvious that the exact amount of some fatty acids could alter according to different culture conditions, which was similar to that presented by Miao and Wu [35]. It is worthwhile to note that C18:1 increased from 30.04% to 36.27% in the optimized medium, which improved the feasibility for producing biodiesels by *Scenedesmus* sp. Additionally, it is observed that the microalga, *Scenedesmus* sp., had a high percentage of α-linolenic acid (12.52% of the total fatty acid), which played an important role in human health [36].

The exact amount of each lipid class was detected under the original and optimized conditions. As shown in Table 6, regardless of whether the medium was optimized, the neutral lipid was a major class (over 80% of total lipids), which was known as the most significant component of biodiesel production. The content of the neutral lipid was remarkably higher than that examined in most of the algal strains [7,12]. Guckert and Cooksey [37] have detailed high pH-induced TAG accumulation in a *Chlorella* species. Depending on this, Gardner *et al*. [32] further proposed that the addition of bicarbonate may be a trigger to promote TAG accumulation. In this study, the value of the pH measured by a pH meter exceeded 11 after cultivation for 9 d, which could resulted in the accumulation of the neutral lipid. Additionally, when the pH value of the growing medium was maintained at 9, the content of the neutral lipid was significantly reduced in the preliminary trial. Therefore, we speculated that high pH stress may result in neutral lipid accumulation in *Scenedesmus* sp. For *Scenedesmus* sp., further experimentation is required to clearly understand if high pH and bicarbonate addition are involved in TAG accumulation.

**Table 6:** Lipid class analysis of *Scenedesmus* sp. (a) in the original medium; (b) in the optimized medium.

Lipid class	Composition (wt% of total lipids)	
	a	b
Neutral lipid	81.29 ± 0.65	82.32 ± 0.89
Glycolipid	12.56 ± 0.53	10.93 ± 0.47
Phospholipid	6.14 ± 0.35	6.74 ± 0.76

## 3. Experimental Section

### 3.1. Strain and Culture Conditions

The *Scenedesmus* sp. was found as a contaminant in an outdoor cyanobacterium *Plectonema* sp. culture with a highly alkaline environment, then was isolated and purified by the streak plate method. The alga was cultured in a modified soil extract (SE) medium composed of the following components: NaHCO_3_, 2 g L^−1^; NaH_2_PO_4_·2H_2_O, 0.05 g L^−1^; NaNO_3_, 0.5 g L^−1^; CaCl_2_, 0.02 g L^−1^; MgSO_4_·7H_2_O, 0.05 g L^−1^; KCl, 0.1 g L^−1^; A_5_ solution, 1 mL L^−1^; EDTA-Fe^3+^, 1 mL L^−1^; soil extract, 1 mL L^−1^. The A_5_ solution was composed of the following compositions (g L^−1^): H_3_BO_3_ 2.86, MnCl_2_·4H_2_O 1.80, ZnSO_4_·7H_2_O 0.22, CuSO_4_·5H_2_O 0.08 and Na_2_MoO_4_·2H_2_O 0.39. The cultures were incubated in 500-mL Erlenmeyer flasks with 300 mL of culture media at 24 ± 1 °C and illuminated by fluorescent lamps for 24 h (120 µmol m^−2^s^−1^). In all experiments, triplicate batch cultures were set up for each treatment. After cultivation for 9 d, cells were harvested by centrifugation (3000× *g*, 5 min).

### 3.2. RSM Experimental Design

In order to obtain the maximization of lipid production by *Scenedesmus* sp., the optimization of medium components were divided into the following two parts.

#### 3.2.1. Plackett–Burman Design

The Plackett–Burman design is a mathematical approach for identifying the critical factors that influence the response [38]. In the experiment, Plackett–Burman design was utilized to evaluate the significance of each medium component towards lipid production. The chosen variables were NaHCO_3_, NaH_2_PO_4_·2H_2_O, NaNO_3_, CaCl_2_, MgSO_4_·7H_2_O, KCl, A_5_, EDTA-Fe^3+^ and soil extract. Each independent variable was set at two levels: −1 for a low level and +1 for a high level, according to the Plackett–Burman design and preliminary trials. For estimating the experimental error, two dummy variables, whose effects were negligible under high and low concentrations, were employed and twelve experiments run in all. The actual factor levels corresponding to the coded factor levels, together with the experimental data, were given in Table 1. The lipid production, which was the average of the triplicates in each trial, respectively, was considered as the response variable.

#### 3.2.2. Box–Behnken Design

The optimum levels of variables, having significant effects on lipid production, were identified using the RSM based on the Box–Behnken design [39]. The chosen variables were as follows: NaHCO_3_, NaH_2_PO_4_·2H_2_O and NaNO_3_. For this procedure, seventeen experiments, including five replicates of the center points and twelve star points, were required. The levels of NaHCO_3_, NaH_2_PO_4_·2H_2_O and NaNO_3_ were varied as shown in Table 3, while the concentrations of other components were constant, as described above. The predicted value of optimum lipid production and culture conditions were obtained. According to experimental results, the second-degree polynomial equation was as follows, which could calculate the predicted value of lipid production:

Y = a_0_ + a_1_A + a_2_B + a_3_C + a_4_AB + a_5_AC + a_6_BC + a_7_A^2^ + a_8_B^2^ + a_9_C^2^

where A, B and C stand for three variables having significant effects on lipid production, Y represents the value of lipid production, a_0_ is the intercept and a_1_–a_9_ are the estimated coefficients. The Design-Expert program (8.05 version) was utilized to analyze the data obtained. According to the determination coefficient (*R*^2^) and *F*-test, the adequacy of the model was evaluated.

To verify the model of the response surface, the experimental value, obtained under the optimum condition, was compared with the predicted value of the lipid production.

### 3.3. Biomass Determination and Lipid Extraction

In all experiments, three series of batch cultures were set up in parallel for each treatment. Algal biomass was measured by dry weight. Aliquots of 20-mL microalgal suspension were filtered by preweighed GF/C filter paper (Whatman, Poole, UK). The filter paper with biomass was then dried at 105 °C to a constant weight. After cooling down to room temperature, the filter paper was weighed. After cultivation for 9 d, cells were harvested by centrifugation (3000× *g*, 5 min). Intracellular lipid was extracted, as previously reported by Khozin-Goldberg *et al*. [40]. A mixture of methanol-dimethyl sulphoxide, diethyl ether and hexane (1:1:1, v/v/v) were used to extract the lipids. When the algae debris was removed, water was added into the organic solvent loaded with lipids, forming a liquid-liquid separation state. Finally, the upper layer, including diethyl ether and lipids, was transferred into the weighed vial and dried by a stream of N_2_. Lipid production was calculated from the relation: Y= m_t_ × L_t_ (Y, lipid production/g·L^−1^; m_t_, biomass concentration/g·L^−1^; L_t_, lipid content/% dw). The extracted lipids were stored at 4 °C to further analyses.

### 3.4. Lipid Analysis and Fatty Acid Composition

The fatty acid composition of the lipids was analyzed by GC-MS with an Omegawax 250 polyethylene glycol capillary column (length, 30 m; diameter, 0.25 mm; 0.25-µm film thickness) using the method reported by Khozin-Glodberg *et al*. [40]. One microliter samples were injected into the capillary column with a split ratio of 5:1. Helium was employed as the carrier gas with a flow rate of 1.5 mL/min. The temperatures of the injector and detector were both maintained at 250 °C. The column temperature was programmed from a 130 °C at 5 °C/min ramp rate to 250 °C maintained for 5 min. The content of each component was determined according to the area normalization method.

Lipid class separation was performed by silica gel column chromatography, according to the method illustrated by Christie [41]. Typically, the samples of lipids re-suspended in chloroform were loaded onto a silica gel column (Agela, Tianjin, China). Neutral lipid, phospholipid and glycolipid were successively eluted using chloroform, acetone and methanol, respectively. Each component was dried by a stream of N_2_ and then weighed.

## 4. Conclusions

The response surface methodology was employed to optimize the medium compositions for maximal lipid production. The medium containing 3.07 g L^−1^ NaHCO_3_, 15.49 mg L^−1^ NaH_2_PO_4_·2H_2_O and 803.21 mg L^−1^ NaNO_3_ was considered as the optimal medium, which improved lipid production by 54.64% more than that obtained in the initial medium. Profiling of lipids achieved in the optimum medium showed that neutral lipid was the major lipid (over 80% of the total lipid), and the dominant components of the fatty acids were C16:0 and C18:1, suggesting that *Scenedesmus* sp. can be suitable for biodiesel production. Moreover, these results proved that the response surface methodology was useful for enhancing the lipid production of the microalga, *Scenedesmus* sp.

## Supplementary Files

Supplementary File 1 Supplementary Information (PDF, 117 KB)
