# Supplementary material for: Optimization of Medium Using Response Surface Methodology for Lipid Production by Scenedesmus sp
Source: Mar Drugs. 2014 Mar 6;12(3):1245–57. doi: 10.3390/md12031245 (PMC3967207; doi:10.3390/md12031245)
Supplement: Supplementary File 1 — Supplementary Information (PDF, 117 KB) [file marinedrugs-12-01245-s001.pdf]

Supplementary Information

Table S1. Results and experimental layout of *Scenedesmus* sp. in Plackett-Burman design.

Run	X_1	X_2	X_3	X_4	X_5	X_6	X_7	X_8	X_9	Biomass (g L^{-1})	Lipid content (% dw)	Lipid production (mg L^{-1})
1	4	200	25	1000	40	100	0.5	0.5	0.5	0.77	29.92	230.38
2	1	200	100	250	40	100	2	0.5	0.5	0.64	27.23	174.27
3	4	50	100	1000	10	100	2	2	0.5	0.73	25.83	188.56
4	1	200	25	1000	40	20	2	2	2	0.65	32.13	208.85
5	1	50	100	250	40	100	0.5	2	2	0.55	27.79	152.85
6	1	50	25	1000	10	100	2	0.5	2	0.63	31.68	199.58
7	4	50	25	250	40	20	2	2	0.5	0.77	27.97	215.37
8	4	200	25	250	10	100	0.5	2	2	0.79	27.14	214.41
9	4	200	100	250	10	20	2	0.5	2	0.72	24.06	173.23
10	1	200	100	1000	10	20	0.5	2	0.5	0.61	27.24	166.16
11	4	50	100	1000	40	20	0.5	0.5	2	0.71	26.60	188.82
12	1	50	25	250	10	20	0.5	0.5	0.5	0.64	30.72	196.58

X_1—$NaHCO_3$ (g L^{-1}); X_2—KCl (mg L^{-1}); X_3—$NaH_2PO_4 \cdot 2H_2O$ (mg L^{-1}); X_4—$NaNO_3$ (mg L^{-1}); X_5—$CaCl_2$ (mg L^{-1}); X_6—$MgSO_4 \cdot 7H_2O$ (mg L^{-1}); X_7—EDTA-Fe^{3+} (mL L^{-1}); X_8—A_5 (mL L^{-1}); X_9—Soil extract (mL L^{-1}).

As shown in Table 1, $NaH_2PO_4 \cdot 2H_2O$ was the most important variable impacting lipid production with *p*-value less than 0.0001, which accounted for 70.71% of the total contribution. The biomass production was increased with the increasing of phosphate concentration, which accounted for 11.40% of the total contribution. With decreasing phosphate concentration from 100 mg L^{-1} to 25 mg L^{-1}, the cellular lipid content in microalgae *Scenedesmus* sp. increased evidently, where *p* value was less than 0.001 and the contribution of phosphate was 55.19%. Additionally, although the lipid production was increased with the increasing the nitrogen level, the contribution of $NaNO_3$ was low with 4.47%. Finally, increasing the concentration of carbon could promote dramatically growth rate of *Scenedesmus* sp. (*p* lower than 0.0001), which accounted for 80.35%. The lipid production was also improved considerably with increasing carbon concentration, which accounted for 18.27% of the total contribution.

© 2014 by the authors; licensee MDPI, Basel, Switzerland. This article is an open access article distributed under the terms and conditions of the Creative Commons Attribution license (http://creativecommons.org/licenses/by/3.0/).
